# Childhood diarrhoea: a cross-sectional survey on maternal knowledge, hygienic practices and use of oral zinc for home management in a Nigerian community

**DOI:** 10.11604/pamj.2022.42.123.33829

**Published:** 2022-06-15

**Authors:** Ifeoma Peace Okafor, Olufunsho Tope Akinyemi, Barine Nene Wika-Kobani, Tope Olubodun, Ugochukwu Timothy Eze

**Affiliations:** 1 Department of Community Health and Primary Care, College of Medicine, University of Lagos, PMB 12003 Lagos, Lagos, Nigeria,; 2 Department of Community Health, Lagos University Teaching Hospital, Lagos, Nigeria

**Keywords:** Childhood diarrhoea, oral zinc, knowledge, practice, home management, oral rehydration solution, Nigeria

## Abstract

**Introduction:**

diarrhoea is the second leading cause of morbidity and mortality in young children. The aim was to assess maternal knowledge, hygienic practices and home management (HM) of diarrhoea with oral rehyrdation therapy (ORT) and oral zinc in children aged 6-23 months in western Nigeria. Predictors of good knowledge and practice were also assessed.

**Methods:**

this was a community based analytic cross-sectional study. Multistage sampling was used to select mothers of children 6-23 months of age. Data were collected using pre-tested, interviewer administered questionnaires and analyzed using SPSS version 20. Bivariate analysis and multiple logistic regression for predictor variables were done. Level of significance was set at 0.05.

**Results:**

three hundred and seventy one (371) respondents were interviewed (mean age 30.4 ± 5.02 years). 305 (82.2%) had good knowledge of diarrhoeal diseases, 208(56.1%) had good knowledge of home management of childhood diarrhoea, 274 (73.9%) had good maternal hygienic practices and 161 (61.2%) of the 263 mothers who had managed diarrhoea in their children, had good practice. Only 34 (12.9%) of them used zinc tablets and 11 (32.4%) did not complete the full course. Maternal age 30-39 years predicted good knowledge (AOR 3.19 CI 2-6.05). Predictors of good home management practices were: maternal age 30-39 years (AOR 2.78 CI 1.44-5.37), >40 years (AOR 5.55 CI 1.54-20.01) and younger age of the index child, 6-11 months (AOR 4.83 CI 2.29-10.18).

**Conclusion:**

mothers had poor knowledge of the role of zinc supplementation in childhood diarrhoea and use of zinc tablets for diarrhoea was very low. Community based health education should be carried out.

## Introduction

Diarrhoea is one of the childhood preventable and treatable diseases. It is one of the leading causes of death among children less than five years. Nearly one in five child deaths is due to diarrhea [1]. A risk factor for diarrhoea diseases is the hygiene practice of mother/ care givers because hygienic practices play an important role in the prevention of infectious diseases. Most transmissions occur in domestic domain which is the child´s principal habitat thus changes in hygienic behaviours can prevent diarrhoeal diseases [2].

Oral rehydration therapy was introduced in 1975 and it has reduced mortality from diarrhoea significantly [3]. Oral rehydration solution (ORS) is a mode of treatment which is cheap, acceptable, affordable, safe and can be used in any environment [3]. It has been the cornerstone of management of diarrhea in order to prevent diarrhea-related morbidity and mortality in developing countries but there has been little progress towards this trend in the last decade [3]. Since this treatment can be successfully applied at home, it is important that mothers should have the knowledge of uses and preparation at home [3]. Zinc supplementation could help reduce the duration and the severity of diarrhea [4,5] and therefore has an additional benefit over ORS in reducing child mortality [4]. Zinc deficiency is also prevalent among young children in developing countries that have a poor diet and high exposure to gastrointestinal parasites. It is associated with a dysfunctional immune system, growth retardation, and a high risk of morbidities, such as diarrhoea and acute respiratory infections and, subsequently, is responsible for 14% of diarrhoeal deaths among children between 6 months and 5 years of age in Latin America, Africa, and Asia [6]. Zinc supplementation during acute diarrhoea is currently recommended by the World Health Organization (WHO) and the United Nations Children's Fund (UNICEF). It could help reduce the duration and the severity of diarrhea [7].

Every year, millions of diarrhea cases progress to severe episodes or death, a high proportion of which occurs in the first two years of life and declines as the child gets older [1,8]. Diarrhoea is more prevalent in developing world largely due to lack of safe drinking water, sanitation and hygiene, as well as poorer overall health and nutritional factors [1]. About 90% of diarrhoeal deaths worldwide are attributed to unsafe water, inadequate sanitation and poor hygiene [9]. In Nigeria, diarrhoea prevalence rate is 18.8% and is one of the worst in sub-Sahara Africa and above the average of 16%. Annually, it accounts for over 16% of child deaths and an estimated 150,000 deaths mainly amongst children under five years [10]. The year 2015 was a target year for achievement of some global health resolutions and commitments yet the set goals were not met. Goal 4 of Millennium Development Goals was to reduce child mortality and target 4 A was to reduce child mortality by two-thirds between 1990 and 2015 [11]. The global health community called out diarrhoea control as critical to achieving Millennium Development Goal 4 (MDG 4) since diarrhoea is the second leading cause of death in under-fives [11]. However, this goal was not met even as the Sustainable Millennium Goals took off in 2015. One of the targets of the Sustainable Development Goal 3 (SDG 3) is to reduce the under-5 mortality to at least as low as 25 per 1,000 live births [7]. Nigeria still has a long way to go. Under 5 mortality rate was 128 deaths per 1000 live birth in the 2013 NDHS though it dropped significantly from 201 deaths per 1000 live births recorded in 2003 and further to 109 by 2015 [11], but we did not achieve two-third reduction of under 5 mortality of MDG 4 which was 64 deaths per thousand [11] and are yet to achieve under 5 mortality of 25 per 1,000 livebirths of the SDG 3. As a leading cause of death in under-fives, diarrhoea control is critical to achieving SDG 3 [7]. A number of studies have documented the existence of relationship between certain behavioural practices at the family level and increased incidence of diarrhoea among children thus primary prevention of diarrhoea through hygiene intervention. This is based on reducing feaco-oral transmission of pathogens and includes improved sanitation and hygiene education. Behavioural factors are important in determining the uptake and sustainable adoption of sanitation and hygienic practices. While water, sanitation and hygiene interventions are potentially highly efficient, their effectiveness in part depend on behavioural change and context [12].

In areas where the prevalence of zinc deficiency or the prevalence of malnutrition is high, zinc may be of benefit in children aged six months or more. In children older than six months, zinc supplementation may shorten the average duration of diarrhoea by around half a day and probably reduces the number of children whose diarrhoea persists until day seven. In children with signs of malnutrition the effect appears greater, reducing the duration of diarrhoea by around a day [7]. Despite the establishment that ORS is the primary reason for the substantial reduction in morbidity and mortality from diarrhoea in children in developing countries, the use of ORS has lagged for many reasons. Often time the role, benefits and method of preparation of ORS are not emphasized by health workers thus mothers do not know the right method of preparation ORS and do not understand the need to give ORS to the child [12]. This study set out to assess maternal knowledge of diarrhoea and its management, their hygienic practices, zinc supplementation in home management of diarrhoea in children aged 6-23 months and predictors of good knowledge and practice in Southwest Nigeria.

## Methods

**Study design and setting:** this was a community based analytic cross-sectional study conducted from May to October 2014. This study was conducted in Osodi-Isolo Local Government Area (LGA) in Lagos State, Southwest Nigeria. It is further divided into two Local Council Development Areas (LCDAs) namely Isolo and Ejigbo. It is a densely populated area with a mix of high and low socio-economic strata. Isolo LCDA has seven wards. Major source of water supply is bore hole and well. Open dumping, burning and controlled tipping are the methods of refuse disposal. There are seven Primary Health Centres and a General hospital in Isolo. Women and children form a considerable portion of the population.

**Study population and eligibility criteria:** study population comprised mothers of young children in the study area. Only mothers whose last child was between 6-23 months of age and who had been resident in the study area were included in the study. Mothers who could not respond to questions appropriately due to illness or temporary visitors were excluded.

**Sample size calculation:** a minimum sample size was calculated using the Cochran formula for descriptive studies


$$N=\frac{Z^{2}pd}{d^{2}}$$

Where Z is the standard normal deviate at 95% confidence level (1.96), ‘p’ represents the proportion of mothers who used SSS at home 31% [13], ‘q’ is 1-p and ‘d’ is the acceptable margin of error (5%). This gave a minimum sample size of 330. Ten percent was added to make up for non-response and incomplete questionnaires and then rounded up to 370 (371 questionnaires administered).

**Sampling methodology:** multistage probability sampling was used to select the respondents who participated in the study. At each stage, simple random sampling technique was employed. First, three wards were selected out of the seven wards, each having about 70 streets. The total sample size was allocated equally to the three selected wards (approximately 124 respondents each). This was followed by the initial selection of a street from each ward from the current list of streets obtained from the LGA. Next was selection of the houses. The total number of houses on each selected street was assessed on the field. The starting point was determined by simple random sampling, and then moved in consecutively higher numbers. The final stage was the selection of eligible respondents from the houses. Only one mother meeting the inclusion criteria was selected from each house. If there were more than one, only one was selected by simple random sampling (balloting) and interviewed. As the interview of respondents progressed, houses on the selected street got exhausted, and subsequently, more streets were selected by simple random sampling. This process continued until the desired sample size was achieved.

**Data collection instrument:** data collection was done with the aid of structured, interviewer-administered, questionnaires considered suitable by face validation done by Community Health Physicians. The questionnaire sought information on the respondents´ sociodemographic characteristics, household water supply and sanitation and details of the index child. Other questions were on knowledge of diarrhoeal diseases in general and prevention in children, knowledge of home management of diarrhoea including the role of ORS and zinc. Lastly, respondents were interviewed on their hygienic practices and their management practices including the use of ORT and zinc with regards to the last diarrhoeal episode in the index child.

### Variables

Independent variables include respondents´ socio demographic and economic characteristics such as age, marital status, educational level, employment status and number of children. Others include household variables such as size, water supply and sanitation, and details of the index child, like age (in months) and sex. Primary dependent variables include those on knowledge of diarrhoeal diseases in general and prevention in children, knowledge of home management of diarrhoea including the role of ORS and oral zinc. Some of these variables on knowledge are: “What is the meaning of diarrhoea?”, “What are the causes of diarrhoea?”, “What is the major complication of diarrhoea?”, “How can childhood diarrhoea be prevented?”, “What can be used/done in the home management of childhood diarrhoea?”, “How is salt sugar solution (SSS) prepared?”, “How is ORS reconstituted?”, and “What are the roles of SSS/ORS and zinc supplementation in the management of childhood diarrhoea?”.

Other dependent variables include those on maternal hygienic practices and their management practices including the use of ORT and zinc with regards to the last diarrhoeal episode in the index child. Some examples are: “How often do you wash your hands with soap and water before feeding the child?”, “How often do you wash your hands with soap and water after use of toilet?”, “How do you dispose your child´s faeces?”, “What do you use to feed your child?”, “Where do you prepare your child´s food?”, “What is the source of your child´s drinking water?” among others. Dependent variables on diarrhoea management practices include: “What method(s) did you use in treating your child at home during the last episode of diarrhoea?”, “If you used zinc tablets, did you complete the course of treatment?”, “If No, why did you stop?”.

Secondary dependent variables on respondents´ overall knowledge and practice were generated from aggregating scores from the primary variables and then graded. Overall knowledge of diarrhoea and home management was assessed in the following domains: knowledge of diarrhoeal diseases, knowledge of home management of diarrhoea, knowledge of role of oral rehydration and knowledge of role of zinc supplementation in diarrhoea management. Each correct response was assigned one point, otherwise zero. Scores were converted to percentage and graded as poor if < 50% and good if ≥ 50% [14]. Maternal hygienic practices were assessed based on their frequency of hand washing and other hygienic behaviour. The domains of behaviour covered were: disposal of human feaces, use and protection of water sources, water and personal hygiene, food hygiene, and domestic and environmental hygiene. Mothers were questioned on their practices of hand washing before feeding, after changing soiled clothing, after defaecation, among others. Responses of ‘always´ attract one point, otherwise zero. Other hygienic practices reported attracts one point each, otherwise, zero. A composite score was obtained, and 50% cut-off was used in grading into good or poor hygienic practices. Overall practice of home management was assessed using recommended practices namely: use of appropriate oral rehydration therapy, continued feeding, appropriate breastfeeding practices and zinc supplementation [15].

**Quality assurance:** three interviewers were recruited and received a one-day training on the objectives of the study, contents of the questionnaire, study process, study tool administration and ethical considerations. The training helped to minimize intra/inter-observer bias. The questionnaires were pre-tested in another LGA among a similar group of women and only slight adjustments were required.

**Data analysis:** data were processed and analyzed using Statistical Package for Social Sciences (SPSS) version 20.0. All questionnaires which were completely filled were analyzed (371). Quantitative data were summarized with mean, median and range and categorical variables with proportions. Chi-square tests were done and then variables were entered into a multiple logistic regression model, to ascertain predictor variables for good knowledge and practice. Level of significance was 5% (two-tailed P ≤ 0.05).

**Ethical consideration:** ethical approval (ADM/DCS/HREC/APP/1971) was obtained from the Health Research Ethics Committee (HREC) of the Lagos University Teaching Hospital. Written informed consent was obtained from respondents prior to administration of questionnaires. Confidentiality was maintained throughout the study.

## Results

**Socio-demographic characteristics:** a total of 371 mothers participated in the study; all were valid for analysis giving a response rate of 100%. Majority 184 (49.6%) were between 30 and 39 years, mean age ± SD of the mothers was 30.41 ± 5.02, 344 (92.7%) were married/cohabiting. Most 360 (97.0%) of the mothers had at least primary school education, and almost one-quarter were unemployed (Table 1).

**Table 1:** socio-demographic characteristics of respondents

Variable (n=371)	Frequency	Percentage (%)
**Age (years)**		
20-29	165	44.5
30-39	184	49.6
>40	22	5.9
**Mean ± SD**	30.4 ±5.02	
**Marital status**		
Single/never married	11	3.0
Married/co-habiting	344	92.7
Separated/divorced/widowed	16	4.3
**Level of education**		
None	11	3.0
≥ Primary	360	97.0
**Employment status**		
Unemployed	98	26.4
Employed	273	73.6
**Tribe**		
Yoruba	169	45.6
Non - Yoruba	202	54.4
**Household size**		
1-3	102	27.5
4-6	236	63.6
≥7	33	8.9
**Median(range)**	4 (1-10)	
**Number of children**		
1-2	239	64.4
2-4	117	31.5
5-6	15	4.0
**Median(range)**	2 (1-6)	
**Age of last child (in months)**		
6-11	155	41.8
12-17	116	31.3
18-23	100	27.0
**Meam ±SD**	13.30± 5.22	
**Sex of last child**		
Male	159	42.9
Female	212	57.1

**Knowledge of diarrhoea:** two hundred and sixty-six (60.9%) of the respondents correctly defined diarrhoea as passage of watery stool more than 3 episodes per day while 136 (36.7%) gave incorrect definition. Concerning the causes, 340 (91.6%) knew that diarrhoea can be caused by contaminated food and water while 192 (51.8%) wrongly attributed it to teething. Majority 209 (56.3%) of the mothers knew dehydration as the main complication of diarrhoea. Three hundred and forty-four (92.7%) were aware of ORS use in home management, 257 (69.3%) knew about SSS and 142 (38.3%) knew they should continue breastfeeding and giving other meals during diarrhoea episodes (Table 2).

**Table 2:** respondents’ knowledge of diarrhoeal diseases and home management of childhood diarrhea

Variable (n = 371)	Frequency	Percentage (%)
**Meaning of diarrhoea**		
Correct	226	60.9
Incorrect	136	36.7
Don’t know	9	2.4
Causes of diarrhoea*		
Contaminated water and food	340	91.6
Poor sanitation	255	68.7
Teething	192	51.8
Sugary food	168	45.3
Normal diet	26	7.0
Over eating	51	13.7
Too much heat	56	15.1
Breastfeeding during pregnancy	86	23.2
Don’t know	8	2.2
**Major complications of diarrhoea**		
Dehydration	209	56.3
Vomiting	72	19.4
Loss of appetite	48	12.9
Don’t know	37	10.0
Others	5	1.3
Methods of diarrhoea prevention*		
Avoidance of contaminated water	337	90.8
Good maternal hygiene	295	79.5
Good Child hygiene	311	83.8
Good environmental hygiene	308	83.0
Prompt treatment of infections	114	30.7
Reducing sugary food intake	143	38.5
Regular use of ORS	137	36.9
Eating well cooked food	159	42.9
Avoiding contaminated food	292	78.7
Immunization (Rotavirus)	89	24.0
Don’t know	4	1.1
Home management*		
Oral rehydration solution	344	92.7
Salt sugar solution	257	69.3
Continuing breastfeeding/other food	142	38.3
Zinc	98	26.4
Antibiotics	172	46.4
Herbs	75	20.2
Don’t know	6	1.6

The major source of information on preparation of ORS/SSS was health worker (76.8 %). Majority 310 (83.6%) knew how to re-constitute ORS while 241 (65.0%) knew how to prepare SSS. About two-thirds 249 (67.1%) of the respondents knew the correct role of ORS/SSS in the management of childhood diarrhoea. About 50% of the mothers did not know the role of zinc in diarrhoea management, 22.6% knew that it reduces severity of diarrhoea, 19.1% know that it reduces the duration of diarrhoea and its role in preventing further occurrences in the ensuing 2-3months was known by only 9.4% of the mothers (Table 3). Majority 266 (82.2%) had good knowledge of diarrhoeal diseases, 208 (56.1%) had good knowledge of home management of childhood diarrhoea, 249 (67.1%) had good knowledge of role of oral rehydration, and 59 (15.9%) had good knowledge of the role of zinc in childhood diarrhoea management. Overall, 270 (72.8%) had good knowledge of diarrhoeal diseases and its management (Table 3).

**Table 3:** respondents’ knowledge of ORS/SSS and their overall knowledge grade

Source of information about ORS/SSS preparation*	Frequency (%)
Health worker	**285 (76.8)**
Friends	**58 (15.6 )**
Family members	**70 (18.9)**
Instructions on ORS packet	**40 (10.8)**
Media	**46 (12.4)**
Others	**1 (0.3)**
**Preparation of SSS**	
Correct	**241 (65.0)**
Incorrect	**24 (6.5)**
Don’t know	**106 (28.6)**
**Re-constitution of ORS**	
Correct	**310 (83.6)**
Incorrect	**5 (1.3)**
Don’t know	**56 (15.1)**
Role of ORS/SSS *	
Prevents dehydration/rehydrates the child	**249 (67.1)**
Stops diarrhoea	**148 (39.9)**
Prevents diarrhoea	**70 (18.9)**
Don’t know	**7 (1.9)**
Others	**33 (8.9)**
Role of oral zinc *	
No effect	**7 (1.9)**
Reduces the severity	**84 (22.6)**
Reduces the duration of diarrhoea	**71 (19.1)**
Prevents dehydration	**42 (11.3)**
Prevents further occurrences in the ensuing 2-3months	**35 (9.4)**
I don’t know	**187 (50.4)**
**Overall knowledge of diarrhoea and home management of childhood diarrhoea**	
**Variable (n=371)**	
**Knowledge of diarrhoeal diseases (meaning, causes, complicatons & prevention)**	
Good	**305 (82.2)**
Poor	**66 (17.8)**
Knowledge of home management of diarrhoea	
Good	**208 (56.1)**
Poor	**163 (43.9)**
Knowledge of role of oral rehydration	
Good	**249 (67.1)**
Poor	**122 (32.9)**
**Knowledge of the role of zinc in diarrhoea management**	
Good	**59 (15.9)**
Poor	**312 (84.1)**
Overall knowledge grade	
Good	**270 (72.8)**
Poor	**101 (27.2)**

**Table 4:** hand hygiene practices

Variable (n=371)	Never (%)	Sometimes (%)	Frequently (%)	Always (%)
Hand wash of child before feeding	56 (15.1)	85 (22.9)	91 (24.5)	139 (37.5)
Maternal hand wash after changing child’s soiled clothing	7 (1.9)	75 (20.2)	77 (20.8%)	212 (57.1)
Hand wash of child after defeacation	48 (12.9)	82 (22.1)	68 (18.3)	173 (46.6)
Maternal hand wash before feeding child	11 (3.0)	93 (25.1)	78 (21.0)	189 (50.9)
Maternal hand wash after defecation	2 (0.5)	34 (9.2)	55 (14.8)	280 (75.5)
Feeding child with left-over food	283 (76.3)	77 (20.8)	7 (1.9)	4 (1.1)
Warming of leftover food (n=88)	10 (11.4)	25 (28.4)	15 (17.0)	38 (43.2)

**Hygienic practices:** washing of child´s hands before feeding was always done by some mothers 139 (37.5%), 173 (46.6%) of the mothers always washed their child´s hands after defeacation, 189 (50.9%) always washed their hands with soap and water before feeding their child, 218 (75.5%) of the mothers always washed their hands after use of toilet (Table 4). Sixty-two percent used water and soap to wash child´s hands before feeding, Majority, 324 (87.3%) of the women used cup and spoon to feed their child and 224 (60.4%) always prepared food on the table. Overall, majority 274 (73.9%) of mothers had good maternal hygienic practices (Table 5).

**Table 5:** food hygiene practices

Variables	Frequency	Percentage (%)
**Items used for washing child hand before feeding**		
Water only	85	22.9
Water and soap	230	62.0
Does not wash child’s hand before feeding	56	15.1
**How child’s feaces is disposed**		
Toilet	94	25.3
Dustbin	148	39.9
Open dumping	9	2.4
Toilet and dustbin	119	32.1
Others	1	0.3
Items used to feed child*		
Cup and spoon	324	87.3
Feeding bottle	71	19.1
Bare hand	19	5.1
**Where child’s food is prepared**		
On the ground	16	4.3
Sometimes on the table	61	16.4
Frequently on the table	70	18.9
Always on the table	224	60.4
**Source of child’s drinking water**		
Tap	38	10.2
Well	8	2.2
Borehole	98	26.4
Bottled water	143	38.5
Sachet water	84	22.6
**Place of storage of child’s drinking water**		
Covered keg	45	12.1
Plastic bucket with cover	97	26.1
Uncovered keg/bucket	2	0.6
Uses bottled/ sachet water	227	61.2
**Overall hygienic practices score**		
Good hygienic practices	274	73.9
Poor hygienic practices	97	26.1

**Home management of childhood diarrhoea:** overall, commonest methods used at home were UNICEF ORS 170 (64.6%); antibiotics 137 (52.1%), SSS 73 (27.7%) and anti-diarrhoeal drugs 49 (18.6%). Only a few administered oral zinc 34 (12.9%). Out of the 34 that used oral zinc, 11 (32.4%) of them did not complete the 10-14 days course. Reasons given for stopping oral zinc include: child got better, unpleasant taste, thought the child was to take fewer than 10 tablets and vomiting/refusal by child. UNICEF ORS was the commonest rehydration solution given 170 (64.6%). During the last episode of diarrhoea, less than two-thirds, 162 (61.6%) of women breastfed more often. Overall, more than one-third 102 (38.8%) had poor practices concerning home management of diarrhoeal diseases (Table 6).

**Table 6:** home management of childhood diarrhoea

Variable	Frequency	Percentage (%)
**Child ever had diarrhea (n=311)**		
Yes	263	70.9
No	108	29.1
Method(s) used at home during the last episode of diarrhoea *(n=263)		
Salt Sugar Solution (SSS)	73	27.7
ORS (Commercial packet)	170	64.6
Diastop (Antidiarrhoea)	49	18.6
Antibiotics	137	52.1
Herbal preparation	42	16.0
Zinc	34	12.9
**Completed course of zinc therapy 10-14 days (n=34)**		
Yes	23	67.6
No	11	32.4
**Reasons for stopping oral zinc (n=11)**		
Child got better	2	18.2
Unpleasant taste/ side effects	1	9.1
Thought the child was to take fewer than 10 tablets	1	9.1
Others	7	63.6
**Rehydration solution used during the last episode (n=263)**		
Salt sugar solution	73	27.8
ORS (Commercial packet)	170	64.6
Water only	10	3.8
None	3	1.1
Others	7	2.7
Feeding practices during the last episode of diarrhoea* (n=263)		
Breastfed more often	162	61.6
Gave usual meals	135	51.3
Stopped adding milk to cereal	61	23.2
Fed minimally to rest bowel	39	14.8
Gave plain dilute pap	79	30.0
Restricted fluid intake	19	7.2
Stopped/reduced breastfeeding	19	7.2
Withheld sugary foods	19	7.2
**Overall practice of home management(n=263)**		
Good	161	61.2
Poor	102	38.8

**Predictors of good knowledge and practice:** on Table 7, multiple logistic regression showed that mothers aged 30 -39 years were about 3 times more likely to have good knowledge of childhood diarrhoea and its home management than mothers aged 20-29 years (AOR 3.48 CI 2.00-6.05). Higher maternal educational level predicted good knowledge (AOR 3.19 CI 1.36-7.47). Older maternal age predicted good home management practices. With age range 20-29 years as reference, older mothers had significantly higher odds of having good practices, 30-39 years (AOR 2.78 CI 1.44-5.37), >40 years (AOR 5.55 CI 1.54-20.01). Younger age of the index child, 6-11 months also predicted good home management practices regarding childhood diarrhoea (AOR 4.83 CI 2.29-10.18).

**Table 7:** factors associated with knowledge of diarrhoea and home management practices

	Overall knowledge of childhood diarrhoea and its home management					
	Poor (%)	Good (%)	p- value	AOR	95% CI	p-value
**Age of mother (years)**	n=101	n=270				
20-29	63 (38.2)	102 (61.8)	<0.001*	1		
30-39	29 (15.8)	155 (84.2)		3.48	2.00-6.05	<0.001*
>40	9(40.9)	13 (59.1)		1.10	0.37-3.26	0.864
**Level of education**						
No formal/Primary	14 (50.0)	14 (50.0)	0.005*	1		
≥Secondary	87 (25.4)	256 (74.6)		3.19	1.36-7.47	0.008*
**Household size**						
1-3	31 (30.4)	71 (69.6)	0.588	1		
4-6	60 (25.4)	176 (74.6)		1.11	0.61-2.02	0.738
≥7	10 (30.3)	23 (69.7)		0.54	0.17-1.74	0.305
**Number of children**						
1-2	67 (28.0)	172 (72.0)	0.463	1		
3– 4	32 (27.4)	85 (72.6)		0.94	0.51-1.74	0.840
5-6	2 (13.3)	13 (86.7)		3.27	0.50-21.49	0.218
**Age of last child (months)**						
6-11	40 (25.8)	115 (74.2)	0.850	1		
12-17	32 (27.6)	84 (72.4)		0.76	0.43-1.36	0.356
18-23	29 (29.0)	71 (71.0)		0.83	0.45-1.55	0.567
**Home management practices regarding childhood diarrhoea**						
	n=102	n=161				
**Age of mother (years)**						
20-29	49 (42.6)	66 (57.4)	0.474	1		
30-39	46 (35.1)	85 (64.9)		2.78	1.44-5.37	0.002*
>40	7 (41.2)	10 (58.8)		5.55	1.54-20.01	0.009*
**Level of education**						
No formal/Primary	9 (47.4)	10 (52.6)	0.425	1		
≥Secondary	93 (38.1)	151 (61.9)		2.01	0.67-6.03	0.213
**Household size**						
1-3	23 (33.8)	45 (66.2)	0.443	1		
4-6	67 (39.4)	103 (60.6)		0.81	0.40-1.67	0.573
≥7	17 (51.5)	16 (48.5)		0.61	0.17-2.25	0.459
**Number of children**						
1-2	54 (32.3)	113 (67.7)	0.017*	1		
3– 4	41 (49.4)	42 (50.6)		0.39	0.20-0.77	0.007*
5-6	7 (53.8)	6 (46.2)		0.24	0.05-1.20	0.082
**Age of last child (months)**						
6-11	21 (21.0)	79 (79.0)	<0.001*	4.83	2.29-10.18	<0.001*
12-17	46 (50.5)	45 (49.5)		0.90	0.46-1.77	0.775
18-23	35 (48.6)	37 (51.4)		1		
**Sex of last child**						
Male	41 (38.7)	65 (61.3)	0.977	1		
Female	61 (38.9)	96 (61.1)		0.98	0.56-1.72	0.941
Overall Knowledge						
Poor Knowledge	23 (38.3)	37 (61.7)	0.935	1		
Good knowledge	79 (38.9)	124 (61.1)		0.61	0.30-1.24	0.173
**Maternal hygienic practices**						
Poor	37 (47.4)	41 (62.6)	0.061	1		
Good	65 (35.1)	120 (64.9)		1.63	0.89-3.01	0.116

* Statistically significant, AOR Adjusted odds ratio, CI Confidence interval

## Discussion

Many of the mothers knew the correct meaning of diarrhoea and could identify episodes of diarrhoea in their children. Similar results were found in the northcentral and Southeast regions of Nigeria [3,16]. But in Benin, South-south region, only few (26%) of mothers who brought their children to the hospital on account of diarrhoea knew the correct definition of diarrhea [17]. In other countries like India and Ethiopia, most mothers also knew the meaning of diarrhoea [18,19].

Contaminated water and food as source of diarrhoea was commonly identified, also in slum areas in India (80%) [18], relatively higher than findings in Enugu, Iran, and Kenya [3,20,21]. The myth of ‘teething’ as a cause of diarrhoea was held by over half of the mothers (51.8%), in sharp contrast to what was observed in the Enugu study (3.9%) [3]. Such myths may have implications for prevention and control. Similar level knowledge in the domain of diarrhoea definition, causes and prevention, was reported in Sokoto, northwest Nigeria [22]. In Iran where health and social services have been disrupted for several years, only 3.7% had a good knowledge [20]. Proportion of mothers with good knowledge about home management of diarrhoea was similar to result from a predominantly urban part of eastern Ethiopia (Diredawa City) [19]. Conversely, much poorer knowledge was observed in studies done in northern Nigeria: Maiduguri (20%), [23] Katsina, Kebbi and Zamfara (<1%) [24]. Despite the health facility location of these studies, the very low literacy level of the respondents which is common in that region, was probably responsible for the very poor knowledge observed.

Though majority of our respondents knew ORS, only about a quarter (26.4%) knew about zinc supplementation. In Northwestern Nigeria, 32% of the over 4,000 caregivers studied had heard of the use of zinc in children but not all (84.3%) knew it was used in the treatment of diarrhea [24]. Less than 50% ORS awareness, and 0% zinc knowledge were recorded in urban slum areas of Aligarh, India where the sanitation is poor and literacy level, very low [25]. In a health facility setting in Port Harcourt, Southern Nigeria, mothers´ awareness of zinc supplementation was equally poor (36.9%) [26]. This low knowledge of zinc is worrisome despite the establishment of its benefit for many years now. Mothers´ knowledge of correct preparation of ORS was fairly high (65.0%) in contrast to reports from other studies [25-27]. This level is still sub-optimal.

Though many respondents knew the actual role of ORS/SSS which is to prevent dehydration, misconceptions about its use to stop or prevent diarrhoea were common. Mothers in Anambra, southeast Nigeria, (87.7%) [28] and Pakistan (95.94%) [29] had better knowledge in this regard. With regards to hygienic practices, low frequencies of child and maternal hand washing always with soap and water before feeding and after defeacation were also reported in rural Vietnam [30] but higher rates in Diredawa, Ethiopia [19]. These sub-optimal hand hygiene practices facilitate the occurrence of childhood diarrhoea, especially as our results showed that overall, maternal hygienic practice was significantly associated with diarrhoea prevalence, thus supporting previous observations [30,31]. ORS use was much higher than SSS in this study, a common pattern in Central America and West Africa [32-34], but not in Kenya [13]. The higher level of usage of ORS might be attributed to its relatively easy re-constitution process. Some mothers used antibiotics for diarrhoeal episodes in their young children. This is a common practice reported in other parts of the country, Africa and beyond: Northern Nigeria (23.6%) [24], Kenya (36.7%) [35] and Dominican Republic (73%) [36], Burkina Faso [33] and Ghana [37]. A systematic review showed that harmful practices in the management of childhood diarrhoea were quite common [38]. Children were usually given other medications like anti-diarrhoeals and herbal mixtures among other things for diarrhoea. In Senegal, only 23.2% of mothers and caregivers interviewed had good management practices [39].

Only about one in ten mothers gave zinc and two-thirds of them completed 10-14 days of the therapy. Probably these few women consulted health workers, and they were eager to see their children recover. In the Port Harcourt study, one quarter of the caregivers used it [26]. In Rarieda, a rural poor area of Western Kenya, 67% used zinc and 84% of them reported taking the full 10-day course [35]. These high rates were recorded following two years of sustained community intervention on zinc treatment at home. In another poor area in Nairobi, Kenya, use of zinc was very low [40]. In northwestern Nigeria, 75.5% adhered to a health centre-supplied 10-day course of zinc treatment for 10 days and major reason for discontinuation were vomiting and refusal by child [24]. This goes in line with results from a systematic review which showed that oral zinc increases the risk of vomiting [4]. In Kenya, none indicated that they stopped because of unpleasant taste or side-effects [40]. Efforts have been on-going to scale-up use of ORS and zinc in Nigeria [6] and a recent programme evaluation showed significant improvements [41].

Older mothers were more likely than younger ones to have good knowledge of childhood diarrhoea and its home management. Probably, the younger mothers do not have as much exposure as the older ones. In the Port Harcourt study, mothers´ age and educational level significantly influenced knowledge [26]. Two separate Ethiopian studies with similar objectives showed no such association [42,43] but in the Addis Ababa location, participants´ occupation was significantly associated with knowledge [43]. The significant role that having younger children played in imbibing good home management practices in our study, may be borne out of greater anxiety for the younger babies. The older children are presumably more resilient due to stronger immunity.

Strengths and limitations of study

Studies of this nature have largely been carried out at health facilities, but this one was done at the community level hence gives a better reflection of scientific evidence. Rigorous sampling methodology and quality assurance mechanisms were deployed for quality data. The study content is quite robust and covered numerous study variables. Due to the study design, causal inferences cannot be made. Face to face interview makes room for possible false claims with regards to practice. In addition, there is higher chance of recall bias which could have been reduced by direct observation. More detailed studies could be conducted to ascertain other factors relevant in the sub-optimal management practices recorded.

## Conclusion

Mothers had good knowledge of diarrhoea, but there was a knowledge gap for the role of oral zinc. Home management practices for childhood diarrhoea were sub-optimal and oral zinc was hardly used. To meet the SDG 3, health education on diarrhoea management (especially on the role of zinc) for mothers at the community level are recommended.

### What is known about this topic

- *Diarrhoea is a common cause of morbidity and mortality among young children in LMICs;*
- *Cost-effective management/interventions include oral rehydration therapy and zinc tablets;*
- *Use of these interventions has lagged over the years*.

### What this study adds

- *Mothers had poor knowledge of oral zinc and its role in diarrhoea management;*
- *Older maternal age and younger age of child predict good diarrhoea management practices*.
